# Gastrointestinal Involvement in Adult IgA Vasculitis: A Comprehensive Review

**DOI:** 10.7759/cureus.104723

**Published:** 2026-03-05

**Authors:** Supritha Chintamaneni, Praveen Wickremasinghe, Batool Mubashar, Monica Multani, Sushovan Guha

**Affiliations:** 1 Internal Medicine, HCA Houston Healthcare Kingwood, Houston, USA; 2 Gastroenterology and Hepatology, Scripps Clinic, San Diego, USA; 3 Gastroenterology, William Carey University College of Osteopathic Medicine, Hattiesburg, USA; 4 Gastroenterology, Larkin Community Hospital South Miami Campus, Miami, USA; 5 Gastroenterology and Hepatology, HCA Houston Healthcare Kingwood, Houston, USA

**Keywords:** corticosteroid and immunosuppressive treatment, gastrointestinal involvement, iga vasculitis (igav), immune complex deposition, prognostic biomarkers

## Abstract

Immunoglobulin A vasculitis (IgAV), formerly Henoch-Schönlein purpura (HSP), is a small-vessel vasculitis with IgA (immunoglobulin A) immune complex deposition that predominantly affects the skin, joints, kidneys, and gastrointestinal (GI) tract. While less common in adults than in children, IgAV (immunoglobulin A vasculitis) is more severe and less well-characterized in adults. GI involvement in adults is associated with significant morbidity, motivating a comprehensive understanding of IgAV pathophysiology, diagnosis, and management. We synthesized current literature on adult IgAV with GI involvement, including its epidemiology, immune mechanisms, genetic drivers, diagnostic approaches, treatment modalities, and prognostic factors. The pathogenesis of IgA vasculitis involves immune complex-mediated small-vessel inflammation driven by aberrant IgA responses, proinflammatory cytokines, genetic predisposition, and environmental triggers. GI involvement occurs in 37-65% of adult IgAV cases, with symptoms ranging from abdominal pain and hematochezia to severe complications such as perforation and ischemia. Diagnosis relies on measurement of laboratory markers (elevated C-reactive protein, Neutrophil-to-Lymphocyte ratio, D-dimer) and imaging (computed tomography, endoscopy). Treatment includes corticosteroids for moderate-to-severe cases, with immunosuppressants (mycophenolate mofetil, intravenous immunoglobulins) reserved for refractory cases. Surgical intervention is rare but necessary in cases of life-threatening complications. Prognostic factors include age, renal involvement, and genetic markers. Adult GI IgAV requires early recognition and a multidisciplinary approach for effective management. Future research should explore advanced biomarkers and therapeutic strategies to improve outcomes.

## Introduction and background

Immunoglobulin A vasculitis (IgAV), formally known as Henoch-Schönlein purpura, is a type of leukocytoclastic vasculitis characterized by bleeding and inflammation of small blood vessels within the skin, intestines, kidneys, and joints [1]. The disease was first described in 1802 by Heberden [2], with further recognition of the correlation of purpura and arthralgias by Schönlein in 1837 [3]. Later, Henoch would contribute the final addition of gastrointestinal (GI) symptoms in 1874 and renal involvement in 1899 [4]. The clinical classic triad consists of palpable purpura, joint symptoms, and abdominal pain [5].

IgAV is the most common systemic vasculitis in children, with an incidence ranging from three to 27 cases per 100,000 annually. By contrast, it is rare in adults, with an incidence of fewer than two cases per 100,000 per year. Its heterogeneous presentation and varying severity often make it more deleterious [6]. While IgAV is well-documented in children, its GI involvement in adults remains relatively rare and less studied. There is a lack of literature specifically addressing the GI manifestations of IgAV in adults. Therefore, this review aims to examine the GI involvement of IgAV in adults through a comprehensive review of the literature.

Immune mechanisms underlying GI involvement of IgAV in adults

An imperative consideration of GI involvement in IgAV is the significant presence of IgA in the lymphoid tissue of the gut. A proposed hypothesis suggests microorganisms activate the innate immune response of digestive mucous membranes, which may dysregulate the digestive lymphoid organs, thereby driving the development of IgAV [7]. Thus, GI infections must be investigated as potential causes of IgAV. Peyer’s patches are the main locations for gut IgA responses, which may explain why GI involvement is more frequent in IgAV compared to other vasculitides [8].

The frequent coexistence of GI and joint manifestations in IgAV suggests a shared cytokine-mediated inflammatory pathogenesis. Prior studies have highlighted a potential role for interleukin-1β (IL-1β) in joint involvement in IgAV, with evidence demonstrating amplified IL-1β secretion in patients with concurrent GI manifestations [9]. Elevated IL-1β activity has also been implicated in inflammatory bowel disease and murine models of colitis, supporting its role in intestinal inflammation [10]. Younger age has been associated with increased IL-1β release in patients with both GI and joint involvement, which may partially explain the more severe disease phenotype observed in this population [11]. Clinically, patients presenting with arthralgias have been shown to exhibit a higher prevalence of GI involvement [12].

In addition to IL-1β, elevated serum interleukin-6 (IL-6) levels have been reported in acute intestinal ischemia and inflammatory joint conditions [13,14]. IL-6 promotes endothelial cell activation through pro-inflammatory signaling pathways, including NF-κB, resulting in increased production of chemokines such as interleukin-8 (IL-8) and monocyte chemoattractant protein-1 (MCP-1). This cascade enhances adhesion molecule expression and facilitates neutrophil recruitment to sites of vascular inflammation [15]. Consistent with this mechanism, patients with GI involvement in IgAV demonstrate distinct neutrophil activation profiles, including elevated neutrophil-to-lymphocyte ratios and increased tissue neutrophil infiltration compared to patients without GI manifestations [16].

Another study reported that in IgAV patients with GI and renal manifestations, higher levels of IL-6 and IL-8 were observed. A separate study observed significantly higher levels of immunoglobulin M (IgM) anti-phosphatidylserine prothrombin (anti-PSPT) for patients with GI involvement [12].

Genetic predisposition and environmental triggers for GI involvement of IgAV in adults

IgAV has a strong genetic susceptibility with its association to human leukocyte antigen (HLA). A relatively recent study confirmed a remarkable association of this vasculitis with the HLA-DRB1*01 (Human Leukocyte Antigen - Discrimination Region Beta, allele 01) genotype and Caucasian individuals [12]. Another small study conducted with 52 patients suggested a protective genetic mechanism against GI involvement in individuals with IgAV who also had a polymorphism at codon 469 of ICAM1 (intracellular adhesion molecule 1) [12].

Patients with severe GI manifestations or with persistent renal involvement did not exhibit any specific HLA-DRB1 (Human Leukocyte Antigen - Discrimination Region Beta 1) association other than the underlying association with HLA-DRB1*01 [17]. Furthermore, the presence of complement component 3 (C3) and vascular IgA deposits in submucosal or chorionic lesions supports the role of immune complex deposition in disease pathology rather than a direct genetic correlation [18].

Environmental factors, such as infection, may trigger IgAV by activating autoimmune mechanisms primarily linked to the HLA-class II region [12]. Infectious agents like group A *Streptococcus*, human parvovirus, and *Giardia* have been implicated in triggering IgAV, with respiratory or GI infections often preceding the onset of symptoms [7,19]. While *Giardia* and *Cryptosporidium* have been suggested as triggers, their individual presence may not be sufficient to cause IgAV; however, a combination of GI infections could contribute to IgA complex formation and deposition [17,20]. *Helicobacter pylori* (Hp) infection has been associated with gastric IgAV on multiple occasions [7,21-23].

Medications such as angiotensin II receptor antagonists, angiotensin-converting enzyme inhibitors, nonsteroidal anti-inflammatory drugs, and antibiotics (e.g., clarithromycin) may serve as inciting agents of IgAV [24-25].

Other triggers of IgAV include hematologic malignancies and solid tumors of the lung and prostate. The prevalence of malignancies in patients with vasculitis is about 5%, of which two-thirds are hematologic malignancies, and one-third are solid tumors. Upon completion of the cancer treatment, resolution of the vasculitis without recurrence occurred while tumor recurrences in some patients coerced subsequent resurgence of the vasculitis, suggesting that the vasculitis is a paraneoplastic syndrome [26].

Other works have suggested that smoking status or the distribution of skin lesions may predict GI involvement of the disease [27].

Epidemiology for the GI involvement of IgAV

GI involvement has been typically reported in 37-65% of adult cases of IgAV [8,28-29], although one very small study reported an incidence of 80% [30]. This wide range of GI involvement may be a result of age differences, as adults under 40 have an increased risk [31]. In addition, GI manifestations are rarely present at the onset of IgAV [32]. In a single-center retrospective study of 417 patients, the prevalence of GI manifestations at the onset of disease was 13.7%. This figure rose to 64.5% when the disease was already established [33]. Blanco et al. reported that in adult IgAV, there was a higher incidence of lower GI involvement at disease onset, but that GI involvement has a similar prevalence between children and adults [8]. Despite this, GI bleeding occurred more frequently in adults (59.1%) compared to children (28.3%) [8].

Hemorrhagic bullous HSP is a rare but severe manifestation characterized by extensive skin necrosis and bullae formation, often associated with systemic involvement. In several studies, it has been reported that renal, GI system (GIS), and joint involvement were more frequently encountered in patients with hemorrhagic bullous HSP [34-37]. In another study, the frequency of GI involvement (34.5%) and renal involvement (72.4%) in patients with HSP was significantly high (p < 0.05), suggesting a strong association with disease severity [38].

## Review

GI manifestations

Spectrum of GI Symptoms in IgAV and Physiologic Mechanisms

The most common IgAV symptoms (in increasing frequency) are vomiting, nausea, diarrhea, hematochezia, and colicky abdominal pain [8,36,39]. These symptoms may arise from bowel ischemia and edema from the vasculitis [40]. Rarely do GI manifestations appear in the presenting symptoms, but they have been reported [41], including GI bleeds (GB) [42-43].

For patients with IgAV, abdominal pain is often epigastric [44]. Submucosal and subserosal hemorrhage from the vasculitis will cause pain and subsequent thrombosis of small blood vessels. Spasmodic abdominal pain was reported as moderate in 86% of patients in one study. However, this abdominal pain can be particularly severe postprandially [45-46].

Approximately 18-52% of the IgAV patients develop GB, accompanied by abdominal pain [24,47]. In adult IgAV patients with IgAV-GB, the incidence rate of lesions in both the lower and upper GI tracts occurred at an incidence rate of 91.7% [48]. About 30% of the IgAV-GB patients test positive for occult blood in a fecal occult blood test, and 1-5% of these patients can progress to intussusception [41,49].

GI hemorrhages in these cases are typically (66% of cases) mild and only detectable by positive fecal occult blood test, although 20% of hemorrhages were reported as severe and life-threatening [50,51]. In adult IgAV patients, GB is associated with a higher incidence of end-stage kidney disease and a lower rate of clinical remission [52].

GI manifestations are proposed to arise through multiple pathophysiologic mechanisms. While edema and hemorrhage previously mentioned can cause GI symptoms, obstruction presumably from ischemia can occur, as can cholecystitis from vasculitis of the gallbladder [45-46,53-55]. Further, abdominal pain and bleeding can be caused by low levels of Factor XIII [56]. A reduction in peristalsis has been observed in patients with IgAV and may be associated with segmental dilatation of the loops of the small intestine with stagnation of intestinal contents [28]. 

While IgAV ulcers most often occur in the small intestine (most commonly the second portion of the duodenum, followed by the terminal ileum), these lesions can occur anywhere throughout the length of the GI tract [45,57]. There are reports of mild liver damage with cholestasis temporally in these patients [29]. A retrospective study involving endoscopic examination of patients with IgAV found the rectum to be the most involved lower GI tract lesion (80%) [44]. However, there have been cases of atypical lesions resembling solid submucosal tumors in patients presenting with IgAV in the intestine [58-59].

Complications

IgAV-related morbidity and mortality in adults are typically from complications of the GI and renal tract [60]. Ileus is reported in these patients 6.5-9% of the time [8,39]. Intussusception occurs in approximately 5% of patients [21,45-46,61-63]. IgAV is also thought to be one of the causes of stricture in the GI tract [58-59]. A single patient, after laparotomy, was found to have patches of gangrenous small bowel; this is the first adult HSP case of intestinal infarction reported [64]. Abdominal compartment syndrome may develop due to edema of the bowel wall [65]. A reported case described a life-threatening clinical course characterized by multiple GI complications, including GB involvement, septic shock secondary to ileal microperforation, and small bowel obstruction caused by an ileal stricture [45]. Small bowel obstruction in this context is thought to arise from vasculitic inflammation and vascular necrosis, leading to progressive luminal narrowing or complete occlusion [66].

Rare but serious GI complications in patients with IgAV include intestinal perforation, acute acalculous cholecystitis, hemorrhagic ascites with serositis, pancreatitis, biliary cirrhosis [67], invagination [68], and multiple comorbidities may further contribute to patient mortality [69]. Other infrequent complications include bowel ischemia or infarction, necrosis, stricture formation, and GI hemorrhage [19,45-46,61-63,70]. The complication of GI perforation represents the main life-threatening complication despite its rare frequency [19,45-46,61-62,70], in particular because it can occur over a wide timeframe [71]. Surgical complications in IgAV predominantly involve the abdomen, reflecting underlying GI tract vasculitis [68,72]. Among these, intussusception represents the most frequently encountered indication for surgical intervention. Other reported complications span a broad spectrum and include acute appendicitis, mechanical bowel obstruction, intestinal ischemia with necrosis, spontaneous bowel perforation, enteroenteric fistula formation, GI hemorrhage, pseudomembranous colitis, IBD, steatorrhea, acute pancreatitis, gallbladder involvement, ileal strictures, and ventral abdominal pathology [65]. In some cases, post-inflammatory ileal stenosis develops following an acute episode, necessitating surgical resection with anastomosis. Less commonly described manifestations include bladder and appendiceal involvement, esophageal stricturing, and spontaneous hemorrhage affecting the right abdominal wall musculature [68,72].

Relapse of IgAV is an imperative complication to consider, although the literature reports conflicting evidence as to its primary causes. Some studies report that clinical response and relapse rates are similar between IgAV patients with GI manifestations, compared to those without, although the presence of GI manifestations at the time of diagnosis may predict relapse [73]. One study, which pooled pediatric and adult cases, demonstrated that joint and GI involvement were the best predictors of relapse [8,73]. Altogether, these conflicting results motivate the need to more definitively define relapse and investigate genetic and other factors.

Differential Diagnosis and Diagnostic Challenges

Establishing a diagnosis of IgAV on the basis of GI manifestations alone is difficult, as no GI features are specific to the disease, and clinical suspicion typically arises only after the development of purpuric skin lesions. Consequently, IgAV is frequently diagnosed later in patients who initially present with predominant GI symptoms.

Below, a few challenging differentials faced by clinicians for IgAV with GI manifestations at onset are mentioned in Table 1.

**Table 1:** Diagnostic challenges in IgAV with GI manifestations IgAV: immunoglobulin A vasculitis, GI: gastrointestinal

Observed sign/symptom	Differential diagnoses
Acute appendicitis presentation (IgAV-induced; first reported case)	Crohn’s disease (as seen in the reported case [74])
Terminal ileitis in IgAV patients	Crohn’s disease, other vasculitides, and infections [62]
Predominant small bowel involvement (especially erosive duodenitis)	Early inflammatory bowel disease versus IgAV [75]
Small bowel disease findings	Infectious enteritis, Whipple disease, infiltrative disorders, other vasculitides, and causes of acute abdomen [44,62,67,76-78]
Unexplained chronic vasculitis (with/without cancer history)	Occult neoplasm [79]
Serious GI manifestations	Consider evaluation for concurrent gastric Helicobacter pylori infection, as its eradication may aid in disease management [80-81]

The overlapping inflammatory involvement of the terminal ileum observed in IgAV and Crohn’s disease suggests a possible shared pathophysiological mechanism, a concept increasingly supported by published reports. Cases have been described in which Crohn’s disease developed later in life following an episode of IgAV, as well as instances of IgAV occurring in patients with Crohn’s disease receiving adalimumab therapy [70]. Additional reports document IgAV with terminal ileitis complicated by thrombotic microangiopathy [19], along with IgAV-associated terminal ileitis accompanied by rapidly progressive glomerulonephritis triggered by pantoprazole exposure [82]. Furthermore, recurrence of IgAV related to Hp reinfection remains a consideration, given reported Hp reinfection rates ranging from 3% to 20% within one to four years after successful eradication therapy [81].

Joint involvement in IgAV is associated with increased GI complications, including life-threatening bleeding and perforation, highlighting the need for vigilant monitoring of patients presenting with either symptom [83,84].

GI vasculitis has been observed in endoscopic biopsies in the small intestine; however, non-specific biopsy findings have also been reported and may be more common [45,85-86]. In an endoscopic study of IgAV patients done by Nishiyama et al., each biopsy taken from each of the 11 patients showed non-specific findings, indicating that biopsy alone may not be sufficient for diagnosis [86].

Diagnosis

Laboratory Investigations

It is crucial to draw blood and identify specific laboratory values in adult patients with IgAV. Table 2 summarizes the key laboratory findings.

**Table 2:** Summary of laboratory findings in adult immunoglobulin A vasculitis (IgAV)

Elevated/positive	Decreased
Neutrophil to lymphocyte ratio, white blood cell count, C-reactive protein (CRP), erythrocyte sedimentation rate (ESR), prothrombin time, activated partial thromboplastin time (aPTT), International Normalized Ratio (INR), D-dimer, serum IgA, fecal occult blood [87]	Serum hemoglobin [88], platelet counts (correlating with GI bleeding) [89-90], Factor XIII levels [91]

There have been significant associations discovered between pre-treatment elevated NLR and the development of GI manifestations [92], particularly in patients with GI bleeding, making NLR a good prognostic marker [47-93]. NLR may serve as a useful biomarker for predicting the development of HSP-GB and uncomplicated recovery without symptom relapse [94].

D-dimer, a product produced by fibrinolysis occurring after vascular wall damage in IgAV, is an independent predictor of GI involvement. Although the specificity of D-dimer is limited by its association with a broad range of disorders [95], this marker may still assist clinicians, particularly in resource-limited settings, in assessing the risk of GI involvement among adults with IgAV [96].

Other miscellaneous, reported laboratory abnormalities include liver biochemistry, urine screening, stool cultures, and skin miR-146a-5p (microRNA-146a, 5-prime strand) expression. Abnormalities in liver biochemistry have been frequently reported during exacerbations of IgAV in adults [28]. Routine urine screening for hematuria and proteinuria (in addition to the previously mentioned screening for occult GB) is warranted in patients with IgAV [97]. A stool culture testing for common GI pathogens such as *Campylobacter* and *Cryptosporidium/Giardia* may prove to be beneficial, as noted by a recent case report [20]. One study suggested decreased skin miR-146a-5p expression indicates more aggressive acute IgAV disease with GI tract involvement [98].

Imaging Modalities

Whitmore and Peterson first illustrated GI involvement in IgAV patients by radiographic images [99], which demonstrate mucosal thickening and filling defects [100-101]. Before the advent of modern CT (computerized tomography) and sonography, barium and water-soluble radiography were the only techniques available for the visualization of GI changes in IgAV [28]. These modalities enabled identification of inflammatory mucosal thickening, small submucosal hematomas with overlying mucosal ulceration, stenotic parietal hematomas associated with reduced compliance of the involved segment, and increased separation between intestinal loops, reflecting bowel wall thickening and localized edema.

Abdominal sonography is useful in patients presenting with abdominal pain as an initial manifestation or during disease exacerbations of IgAV [28]. Doppler sonography further enables assessment of intestinal blood flow and vascular resistance within affected segments [45]. However, because abdominal sonography is technically more challenging and often less informative in adults than in children, CT is generally preferred for detailed evaluation and longitudinal monitoring of abdominal complications in adult IgAV patients [18,102-103]. While CT offers information comparable to sonography, it provides superior anatomic detail and is particularly valuable when sonographic findings require confirmation or when sonography is limited by technical constraints. In addition, CT facilitates visualization of mesenteric fat attenuation, lymphadenopathy, vascular congestion beneath thickened bowel loops, fatty deposition within the distal ileum, and mucosal wall thickening [20,45,73,84]. On CT imaging, bowel involvement is seen as a circumferential, regular, symmetrical wall thickening with a “target appearance” due to mesenteric edema, engorgement of mesenteric vessels, intramural and extramural masses, and nonspecific lymphadenopathy; however, this target appearance is nonspecific [57,102]. For these reasons, CT has become part of standard analysis.

To bolster diagnostic evidence, both CT and GI endoscopy may be recommended. One study suggested that for patients with IgAV and GI involvement, abdominal CT and double endoscopy are required, as both are needed to confirm and characterize GI involvement and to evaluate its severity, which can impact the therapeutic strategy [8]. Sonography and CT are the ideal complements to endoscopy and GI opacification, since they permit direct visualization of the intestinal wall, epiploon, and mesentery [28]. One case report of a patient with IgAV demonstrated that esophagogastroduodenoscopy (EDG), colonoscopy, interventional radiology angiogram, and CT angiography were used, with each modality providing a unique, necessary perspective, indicating the need for a multi-technique approach [104].

Endoscopic Evaluation

While a multi-technique imaging approach is superior to any individual modality for determining diagnosis and prognosis, endoscopy is the most pragmatic method, as it allows direct visualization with biopsy of the GI tract mucosa [44]. With regard to sensitivity, 61% of CT scans taken in patients with IgAV were found to be abnormal, while 87% of endoscopies in patients with IgAV were found to be abnormal [8].

Upper endoscopy: Upper GI endoscopy is indicated due to its ability to identify the site(s) and extent of disease involvement topographically and descriptively, helping to assess prognosis [28,105]. Utilizing upper GI endoscopy, the most characteristic finding of IgAV is severe, erosive duodenitis in the second portion of the duodenum [28,45,77]. Other plausible endoscopic findings may include erythema, ring-like petechiae, ulcers, ecchymotic lesions, strictures, hematoma-like protrusions, or necrotic areas of the digestive wall seen in the gastric antrum, ileum, and colon, in addition to the second portion of the duodenum [57,67,85,106]. Hematoma-like protrusions/hemorrhagic blebs and extensive loss of duodenal villi, while characteristic of IgAV, are rare [45,90]. Of note, there are contrasting reports on the frequency of ulcers, with some studies indicating ulcers are rare [45], while other studies indicate that ulcers in the small bowel are more frequent compared to the rest of the digestive tract [86]. Forty percent (40%) of patients in one study had upper GI bleeding, with endoscopy revealing small hemorrhages in the gastric mucosa [33].

One study found a high rate of coexisting lesions in the upper and lower GI tracts on endoscopy, lack of predictability of lower GI lesions from examining upper GI lesions and vice-versa, and being able to determine the strength of the treatment necessary to ameliorate lesions based on endoscopy; for these reasons, colonoscopy in addition to upper endoscopy, is necessary for patients with IgAV and GB [48].

Lower endoscopy: Colonic and esophageal involvement occurs with much less frequency, but has a similar appearance to the other lesions found at more common locations on endoscopy. In the colon, the lesions may be segmented or diffuse, appearing throughout, though they may be limited to erythema [28]. Lesions in the lower GI tract have been described as the most severe in some studies, with particular descriptions including redness, petechia, multiple ulcers, and nodular change [44]. GI hemorrhage in adults is typically occult, and petechiae-like lesions have been demonstrated on colonoscopy [106]. As previously mentioned, colonoscopy in these cases has demonstrated significant necrosis in the terminal ileum with a clear transition of friable, inflamed mucosa and unremarkable colonic mucosa [20].

Histopathological Confirmation/Biopsy

The purpose of mucosal biopsies is to exclude IBD and other diseases [44]. Immunohistochemical studies of biopsied material of skin can confirm a diagnosis of IgAV, which is particularly useful in patients who present with GI manifestations as their first symptom [28, 107]. Biopsies should be obtained before steroid therapy is initiated, if possible [61]. A neutrophilic infiltrate is commonly noted within upper GI biopsies, whereas lymphocyte counts do not increase in most IgAV patients [61]. Mucosal biopsy yields leukocytoclastic vasculitis of capillaries and postcapillary venules similar to that of a skin biopsy [108]. Moreover, the absence of histiocytes on histopathologic examination has been associated with a higher likelihood of GI tract involvement [109]. In a separate study, the predominant histologic finding was hemorrhage within the lamina propria, with many specimens additionally demonstrating fibrin deposition, red blood cell sludging, and nuclear debris. Eosinophilic infiltration was also observed in several samples. Notably, leukocytoclastic vasculitis was identified in four biopsies, each involving small capillaries located within the lamina propria [61].

Treatment

Supportive Measures and Symptomatic Relief

The designation of bed rest depends on the clinical status of IgAV and must be temporary [110]. In patients with moderate GI involvement, a placebo-controlled study found that ranitidine (a histamine 2 receptor antihistamine) was found to be effective, wherein the duration of abdominal pain and subclinical GI hemorrhage were significantly reduced. Ranitidine also reduced the pain duration by more than 48 hours in patients with GI exacerbations of IgAV [111]. Spasmolytics have also been shown to be a useful adjunct for patients with GI involvement [53,112].

Nonsteroidal anti-inflammatory drugs (NSAIDs) should be avoided in those with GI manifestations, but are generally used to treat major articular manifestations that do not respond to other analgesics [40,53,112], particularly in patients with massive GI hemorrhage due to the antiplatelet aggregating effect of NSAIDs [113].

Primary Definitive Therapy

Oral prednisone remains the first-line therapy for IgAV and is typically initiated at a dose of 1-2 mg/kg daily for one to two weeks, followed by a taper consisting of 0.5 mg/kg daily for one week and then 0.5 mg/kg on alternate days for an additional week. For patients unable to tolerate oral administration, intravenous (IV) methylprednisolone may be used as an alternative [114]. The primary symptomatic relief of abdominal pain and arthralgias occurs within a few days upon initiation of corticosteroids [40]. Multiple studies bolster use of corticosteroids early in disease progression may be successful in treating even serious forms of vasculitis [40,115]. However, other studies indicate reservations in the usage of corticosteroids, suggesting that first-line corticosteroid therapy should be reserved for patients with severe GI involvement. Some studies show the role of steroids in the prevention of GI complications such as GB and intussusception [116]. One study found 95.7% of the patients with IgAV and GI hemorrhage who had upper and lower GI lesions simultaneously responded effectively to corticosteroid therapy [48]. A meta-analysis found that the NLR was significantly higher in patients with HSP compared to healthy controls (SMD = 1.24 vs. 0.63). Similarly, patients with HSP and GI complications (HSP-GCs) had a notably higher NLR than those without (SMD = 2.24 vs. 0.47) [94]. These findings suggest that elevated NLR may be linked to the inflammatory burden in HSP, particularly in those requiring glucocorticoid therapy.

Noncanonical treatment approaches may be indicated in some extreme cases. Corticosteroid pulses (defined as therapy at or above 250 mg prednisone or the equivalent for several days) may be helpful in patients with massive GI hemorrhage and widespread mesenteric vasculitis [54]. Other studies have stated methylprednisolone-specific pulses are indicated in difficult and life-threatening situations, such as risk of GI complications, including GB, mesenteric ischemia, and intussusception [67,117]. Some reports of corticosteroid-resistant cases suggest severe intestinal IgAV may respond preferentially to IV immunoglobulin therapy (IVIg), suggesting IVIg therapy provides an effective alternative to corticosteroids [118]. Intestinal edema, assessed using abdominal sonography, disappeared 24 hours after Ig (immunoglobulin) infusion. Abdominal ultrasonography showed complete resolution of bowel edema within 24 hours after Ig (immunoglobulin) infusion. Separately, adults with severe GI involvement due to IgAV and markedly reduced factor XIII levels demonstrated rapid symptom relief following treatment with virally inactivated factor XIII concentrate [90, 119, 120]. A case report observed the eradication of a tumor-like lesion located in the cecum and resultant fresh melena in the setting of immediate Factor XIII administration [26].

Immunosuppressive Agents and Other Considerations

Refractory GI involvement is defined as persistence of GI symptoms after three days of GCs (glucocorticoids) treatment (2 mg/kg/day) or a relapse of symptoms during the taper regimen [114]. For patients with refractory GI involvement, mycophenolate mofetil 20-30 mg/kg twice daily with subsequent gradual taper over three months may be used [121].

A randomized controlled trial in 54 adults with severe IgAV compared treatment with GCs alone versus GCs combined with cyclophosphamide (CYC). The addition of CYC did not confer a significant advantage over GCs alone, although improved survival was observed in the GCs plus CYC group. After adjustment of the treatment arms, disease severity emerged as the only factor significantly associated with patient survival [122].

Polyclonal IgG administered as IVIg at a dose of 2 g/kg monthly for severe disease or as IMIg at 0.35 ml/kg every 15 days for moderate disease was reported to be effective in managing chronic cutaneous, articular, and GI manifestations in two open prospective studies involving patients with primary IgA nephropathies and Schönlein-Henoch nephritis. In subsequent reports, monthly high-dose intravenous immunoglobulin (2 g/kg) was administered to a small cohort of IgAV patients who failed to respond to corticosteroid therapy. Rapid improvement in abdominal symptoms was observed, in some cases occurring within hours of infusion [123-124]. More recent adult case reports have further supported the efficacy of high-dose IVIg for severe GI involvement in IgAV [125-127].

There are reports of plasma exchange being used in severe GI vasculitides that persisted despite parenteral feeding [128].

In 1995, Reinauer et al. described the first known case of a 21-year-old woman with IgAV and Hp-positive gastritis; after Hp eradication therapy, the clinical manifestations were resolved [7].

For the treatment of GI lesions of IgAV, dapsone was reportedly effective in 10 adult patients; generally, patients had responded within a week of therapy initiation [129-131].

While adults with IgAV and GI involvement rarely (2-4% of cases) require surgical intervention [8,39,132], gut vasculitis cases with significant blood loss, bowel ischemia, or perforation may prompt exploratory surgery [40,50-51,133].

Prognosis

Factors Influencing Prognosis in IgAV

The short-term prognosis of IgAV depends on the severity of the potential acute involvement of the GI tract [7]. For example, adult patients with GB tend to have worse renal outcomes [134]. Colitis associated with vasculitis with subsequent extensive lower GI hemorrhage is an uncommon presentation of IgAV and can be associated with an increased risk of renal involvement [135].

Inflammation is thought to promote the development of age‑associated diseases by driving the production of proinflammatory cytokines, particularly IL‑6 and TNF‑α (tumor necrosis factor alpha) [8]. The intestinal microbiome plays a central role in inflammatory processes, as microbial products can trigger immune activation, and secretory IgA contributes substantially to the regulation and spatial organization of gut microbial communities [136-137]. Alterations in gut microbiota composition and structure have been documented in pediatric patients with IgAV; however, corresponding data in adult populations remain limited [138].

In light of the component of inflammation, age increases the severity and worsens the prognosis of IgAV in adults; poor prognosis has been reported in IgAV patients older than 60, regardless of GI involvement [74,139]. The risk for bowel ischemia (and its devastating complications) is especially increased in older and polymorbid patients [27]. While younger patients have more frequent joint and GI involvement, older patients have more frequent and severe purpura and glomerulonephritis [75].

A strongly depressed level (<60%) of actor XIII was seen in patients with IgAV and severe GI complications [56]. This appears to be linked to a monumental increase in the release of an enzyme that degrades factor XIII by polymorphonuclear neutrophils [28].

A study found there was a significantly decreased risk of developing severe GI complications in patients without the ICAM1 codon 469 K/E (469 lysine/glutamic acid) genotype. Based on these findings, genetic screening for this polymorphism could help identify patients at high risk who may benefit from monitoring and corticosteroid therapy to prevent GI complications [76].

One study demonstrated that generalized purpura, smoking status, and abdominal pain are clinical predictors of severe GI involvement in adults with IgAV [77].

IgAV patients with odontogenic focal infection (OFI) had higher incidences of GI complications than the IgAV patients without OFI, indicating the presence of OFI is a risk factor in the prognosis of IgAV [78].

An increased D‑dimer level, strongly linked to upper GI bleeding, has been suggested as a prognostic indicator in acute GI conditions and may help identify patients who require urgent endoscopic evaluation [140].

Prognosis

IgAV GI involvement generally has a rare mortality rate (0.7-1%) and is typically due to sepsis-related bowel perforation or mesenteric ischemia [8,47]. Deaths have been reported in cases of adult HSP with severe GI involvement [29,64,141-146]. Some patients with GI involvement experience death despite treatment with steroids and immunosuppressants [62,74].

## Conclusions

In adults, GI involvement represents one of the most clinically significant and potentially life-threatening manifestations of IgAV. Although overall mortality remains low, GI complications such as bleeding, ischemia, perforation, and obstruction substantially contribute to morbidity and may adversely impact renal outcomes and overall prognosis. The frequent coexistence of GI and joint manifestations further underscores a shared inflammatory pathogenesis and highlights the need for early recognition and vigilant monitoring. Given the heterogeneity of presentation, the nonspecific nature of endoscopic and radiologic findings, and the overlap with other inflammatory and infectious conditions, clinicians must maintain a high index of suspicion when evaluating adults with abdominal pain and systemic features suggestive of vasculitis. Early risk stratification using clinical predictors and laboratory markers, combined with timely imaging and endoscopic evaluation, is essential to guide therapy and prevent severe complications. Despite growing recognition of GI involvement in adult IgAV, substantial gaps remain in understanding its pathophysiology, optimal management strategies, and long-term outcomes, emphasizing the need for further focused research in this population.
